# Genetic Diagnosis in a Cohort of Adult Patients with Inherited Metabolic Diseases: A Single-Center Experience

**DOI:** 10.3390/biom12070920

**Published:** 2022-06-30

**Authors:** Livia Lenzini, Gianni Carraro, Angelo Avogaro, Nicola Vitturi

**Affiliations:** 1 Department of Medicine-DIMED, University Hospital, University of Padova, 35128 Padova, Italy; livia.lenzini@unipd.it; 2 Nephrology, Dialysis and Transplant Unit, Department of Medicine-DIMED, University Hospital, University of Padova, 35128 Padova, Italy; giannicarraro63@gmail.com; 3 Division of Metabolic Diseases, Department of Medicine-DIMED, University Hospital, University of Padova, 35128 Padova, Italy; angelo.avogaro@unipd.it

**Keywords:** genetic diagnosis, rare inherited metabolic diseases, adult subjects

## Abstract

Inherited metabolic diseases (IMDs) are genetic conditions that result in metabolism alterations. Although research-based Next Generation Sequencing (NGS) testing for IMD has been recently implemented, its application in a clinical diagnostic setting remains challenging. Thus, we aimed at investigating the genetic diagnostic approach in a cohort of adult patients with IMDs referred to our adult metabolic unit. A retrospective analysis was performed collecting demographic, clinical, and genetic data of patients referred to the Adult Metabolic Unit in Padua from November 2017 to March 2022. In total, 108 adult patients (mean age: 33 years ± 17, 55% women) were enrolled in the study, and 83 (77%) of the patients transitioned from the pediatric metabolic clinics. The most prevalent groups of IMDs were disorders of complex molecule degradation (32 patients) and disorders of amino acid metabolism (31) followed by disorders of carbohydrates (26). Molecular genetic diagnosis was reported by 69 (64%) patients, with the higher rate reported by patients referred from specialty other than pediatric (88% vs. 55%). Almost all the subjects (92%) with disorders of complex molecule degradation had a genetic diagnosis. Patients with disorders of amino acid metabolism and disorders of carbohydrates had almost the same rate of genetic test (39% and 38%, respectively). Among the patients without a genetic diagnosis that we tested, two novel mutations in disease-associated genes were detected. In our single-center cohort, a consistent proportion (36%) of subjects with IMDs reaches the adulthood without a genetic demonstration of the disease. This lack, even if in some cases could be related to disease-specific diagnostic approach or to different disease onset, could be detrimental to patient management and impact to some of the specific needs of adult subjects.

## 1. Introduction

Inherited metabolic diseases (IMDs) are genetic conditions that result in alterations of metabolism. Patients with IMDs have a defective gene that causes an enzyme deficiency. In most IMDs, a single enzyme is either not produced by the body at all or is produced in a non-functional form. Depending on the role of the altered enzyme, its absence could cause an accumulation of metabolites or the absence of essential substances [1]. 

There are hundreds of different genetic metabolic disorders, and their symptoms, treatments, and prognoses vary widely. Moreover, clinical signs can differ even among individuals with similar genotypes, making accurate diagnosis particularly difficult if based only on clinical features [2]. This is why, besides clinical examination, diagnostic work-up is usually complex and involves biochemical and/or enzymatic testing and, less often, molecular investigations [3,4,5,6]. 

In addition to severe neonatal forms, many IMDs can have mild forms with first clinical signs starting in adolescence or very late in adulthood. The number of adult patients with an identified IMD is predicted to increase because of a higher awareness of clinicians to the late onset manifestations and because the number of IMDs is constantly expanding, with new disorders and disease mechanisms being described regularly [1]. 

Genetic testing plays a major role in the management of adult subjects with an IMD as it not only increases the accuracy of diagnosis, but also allows for family members testing and genetic counseling prior to procreation. 

However, these rare genetic diseases have been refractory to traditional gene discovery approaches for the heterogeneous phenotypes and causes and the availability of only a small number of patients to characterize the molecular features of a specific IMD. 

The recent implementation of research-based Next Generation Sequencing (NGS) testing for IMDs has, in part, overcome these difficulties and it is no longer cost prohibitive to use NGS to understand the genetics of each single patient. In fact, the rate of genetic confirmation is becoming higher for all rare genetic conditions, due to the improvement of techniques, which reduced costs and times of report. 

Moreover, nowadays, genetic diagnosis of an IMD can be addressed, in most cases, using targeted (gene panels) sequencing as the biochemical markers allow us to circumstantiate the genetic testing, limiting it to very few genes. Whole-exome and -genome NGS analyses [7,8] are required only in the few cases where these markers are not clearly suggestive of a specific IMD.

However, NGS application to IMDs in a clinical diagnostic setting remains challenging.

Thus, we aimed at investigating the genetic diagnostic approach of adult patients with IMDs in a cohort of subjects referred at our adult metabolic unit.

## 2. Materials and Methods

A retrospective analysis was performed collecting demographic, clinical, biochemical, and genetic data of patients referred to the Adult Metabolic Diseases Center of the University Hospital of Padova from November 2017 to March 2022. The study was approved by the local ethics committee (Approval number: 35324). The electronic medical records were reviewed by two independent operators to collect the age and sex of the patient, reason(s) for referral, the patient’s medical history, and family history. The diagnostic work-up and genetic laboratory investigations were recorded, and if a genetic diagnosis was made, the specific diagnosis was noted. Diagnoses were classified as “clinical”, “biochemical”, “enzymatic”, and “genetic”.

No exclusion criteria were applied. 

Inherited metabolic diseases were categorized following the International Classification of Inherited Metabolic Disorder (ICIMD) group [9].

## 3. Results

### 3.1. General Characteristics and Genetic Diagnosis Rate of the Whole Cohort

In total, 108 adult patients (mean age: 33 years, ranging from 17 to 80 years) with a clinical and biochemical diagnosis of IMD, including 59 women (55%), were enrolled in the study. Of the cohort, 83 (77%) of the patients transitioned from the pediatric metabolic clinics. 

The most prevalent groups of IMDs were disorders of complex molecule degradation (32 patients, 30%) and disorders of amino acid metabolism (31 patients, 29%) followed by disorders of carbohydrate (26 patients, 24%).

**Figure 1:**
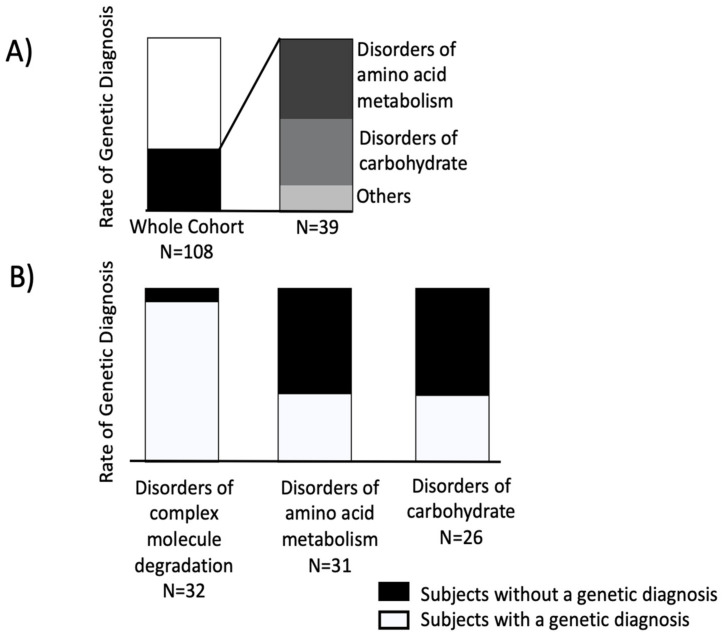
Panel (**A**). Rate of genetic diagnosis in the whole cohort (n = 108) and distribution of disorders in subjects without a genetic diagnosis, according to the ICIMD group classification. Panel (**B**). Rate of genetic diagnosis in the most represented IMD categories of the study cohort.

Molecular genetic diagnosis was reported by 69 (64%) patients (Figure 1, panel A), and 29 (42%) of them were tested in adulthood.

The subjects with the higher rate of genetic diagnosis were those referred from specialty other than pediatric (88% vs. 55%). 

Among the 39 patients without a genetic diagnosis, 18 (46%) were affected by disorders of amino acid metabolism and 15 by disorders of carbohydrate (38%) (Figure 1, Panel A). The remaining subjects had various disorders, such as other disorders of complex molecule degradation, disorders of sphingolipid degradation, disorders of cobalamin metabolism, and disorders of zinc metabolism.

### 3.2. Genetic Diagnosis in Single IMD Categories

To get insights on the rate of genetic diagnosis in single IMD categories, we focused on the three most represented in our cohort (Table 1).

The disorders of complex molecule degradation was the most frequent category in our cohort and patients with Anderson–Fabry disease (OMIM: 301500) were the majority (28 patients, 88%). The cohort includes 15 females and the mean age at the time of the first visit is 34 years, ranging from 16 to 80 years (Table 1). 

Almost all these subjects (30 patients, 92%) (Figure 1, Panel B) had a genetic diagnosis at the time of referral to our center and this was usually performed during familial screening using gene-targeted (i.e., *GLA* gene) approach. In fact, these patients are often members of large family pedigrees of carriers who are referred to our adult metabolic center. Only five subjects transitioned from the pediatric metabolic clinics.

The second most represented category of diseases was the disorders of amino acid metabolism with 31 subjects (Table 1) who were mainly (14 patients, 45%) affected by Urea cycle disorders and inherited hyperammonemias; in this class, the Argininosuccinate lyase deficiency was the most frequent disease (7 patients, 50%) (OMIM: 207900). Female subjects were 12 and mean age at referral was 34, ranging from 16 to 39. Only 39% of patients received a genetic diagnosis (Figure 1, Panel B).

Interestingly, this rate was the same (38%) (Figure 1, Panel B) of subjects belonging to the third category (disorders of carbohydrate). This group (Table 1) comprised almost only disorders of glycogen metabolism (14 patients, 54%) and disorders of galactose and fructose metabolism (11 patients, 42%), with the glycogen storage disease 1a (8 patients, 57%) (OMIM: 232200) and the hereditary fructose intolerance (7 patients, 64%) (OMIM: 229600) being the most common diseases. The mean age of this group of patients was 34 and ranged from 18 to 64; there were 17 females.

### 3.3. New Genetic Diagnoses

Of the adult IMD patients without a genetic diagnosis, seven were tested by us with either whole-exome or gene panel NGS approaches. One carried a mutation on the *GBA* gene, causing Gaucher disease (OMIM 230800), two siblings were mutated in the *ASL* gene causing Argininosuccinate lyase deficiency, and one carried a mutation of the *G6PC* gene causing Glycogen storage disease Ia. All these diagnoses were performed using gene-targeted NGS approaches as the patients’ biochemical markers were suggestive of the causative gene. 

In one subject presenting with a clinical suspicion of disorder of carbohydrate, no disease-causing gene was detected after NGS analysis of the whole exome, while two never described variants in disease-associated genes were detected in the last two cases. The first subject was clinically diagnosed with a Leigh-like syndrome soon after birth and reached the 34 years without a genetic demonstration of the disease [10]. Using NGS analysis through a custom-made targeted mitochondrial panel of 299 genes (Agilent, Milan, Italy), we identified a novel variant (c.223G > C; p.Gly75Arg) of the gene *MRPS34* associated with combined mitochondrial oxidative phosphorylation (OXPHOS) system deficiency predicted by the bioinformatics tools available online (SIFT, Polyphen2, MutationTaster, PROVEAN, VEST3, MutPred, MutationAssessor) to have a deleterious or probably deleterious effect on the functionality of the protein.

The second subject was clinically diagnosed at infantile age with nonketotic hyperglycinemia (disorders of glycine and serine metabolism), but only at 18 years a compound heterozygosity of the *GLRX5* gene was identified by using an NGS whole-exome approach (data not yet published).

## 4. Discussion

In this single-center cohort of adult subjects with a clinical and a biochemical diagnosis of IMD, a consistent proportion of cases (36%) was found to lack the genetic demonstration of the disease. 

However, considering some single IMD categories according to the recent classification of the ICIMD group [9], this rate varied among them, suggesting that heterogeneity could be related to disease-specific diagnostic *iter* and onset. 

In fact, the impact of diagnostic approach is evident considering the category with the higher rate of genetic testing (disorders of complex molecule degradation), which in our cohort is mostly represented by Anderson–Fabry disease (AFD). The diagnosis of this disease is typically performed by enzymatic and genetic testing [11] and often results from screening large series of subjects with phenotypic traits commonly observed in AFD. Moreover, the genetic testing is mandatory to confirm the diagnosis in female subjects, who may present with a normal residual enzymatic activity.

However, the identification of the AFD-causing mutation is also fundamental to select the best treatment solution as the patient’s genotype determines a different response to specific treatments (enzyme replacement therapy vs. chemical chaperone therapy) and is often associated with damage to specific organs [12] which can require adjuvant targeted treatments [13].

Thus, the diagnostic approach to AFD can offer a positive example on how implementing genetic diagnosis by screening of at-risk adult populations and by a systematic testing of family members [14] can successfully lead to a better stratification of patients, in terms of therapeutical choices and prognosis. 

On the other hand, the age at the time of the diagnosis seems to impact on the genetic testing rate of the other two IMDs categories considered (disorders of amino acid metabolism and of carbohydrate) in our cohort. Interestingly, the genetic diagnosis rate is equal in these two, even if they comprise very different disorders, with very heterogeneous clinical and biochemical manifestations. However, they share, for the majority, the age of the onset (neonatal or infantile) and, in fact, all the subjects with these disorders included in this study transitioned from the pediatric metabolic clinics. On the other hand, subjects with disorders of complex molecule degradation were almost all referred from specialty other than pediatric.

These results, even if limited to our single-center experience, raised the issue that there is a consistent proportion of subjects with an IMD diagnosed in infancy who reach the adulthood without knowing the genetic cause of their disease. This can be in part due to the fact that in IMDs, differently from other genetic conditions, the biochemical diagnosis, based on enzymatic activity and/or altered blood and urine metabolites, can be highly predictive of causative condition. 

However, this lack impacts not only the accuracy of the diagnosis, but also some specific needs of adult subjects, such as family members testing and prenatal diagnosis. In fact, as approximately 80% of the IMDs are supposed to be inherited in an autosomal recessive manner [15], genetic consultation should be encouraged in adult subjects with these disorders and their families/partners and pre-conception or pre-natal or pre-implantation genetic tests should be offered. 

Moreover, as the cost of NGS has dramatically dropped, it is no longer cost prohibitive to use NGS to retest every rare disease patient, whose disease-causing gene was not previously recognized, to obtain a better understanding of the genetics of their conditions. This will enable stratification of patients by new genotypes and, possibly, will offer the possibility of new targeted disease-modifying treatments developed in the last years.

In conclusion, considering also that the impact of the neonatal screening on the IMD diagnosis will radically change the actual situation allowing for a very early genetic confirmation in a growing number of IMDs, the next challenge in the diagnosis of IMD will be the implementation of NGS techniques in this emerging field.

## 5. Limitations of the Study

This study is limited to a single-center experience and not all IMDs are represented in our cohort, which could significantly bias the interpretation of the results and limit their extension to the whole group of IMDs. In particular, adult patients with Phenylalanine hydroxylase deficiency (OMIM: 261600), which is the most frequent IMD, were not considered as they were followed at a parallel dedicated clinic, while, on the other hand, the AFD was over-represented in our cohort.

## Figures and Tables

**Table 1:** Characteristics of the three most represented groups of IMDs in the cohort of patients.

Category of IMDs	Gender (F/M)	Age (Range)	Group of IMDs	N of Patients (%)	N of Patients with Genetic Diagnosis (%)
**Disorders of complex molecule degradation**	15/17	34 years (16–80)	Disorders of sphingolipid degradation	29 (91)	30 (90)
			Disorders of glycosaminoglycan degradation	1 (3)	
			Other disorders of complex molecule degradation	2 (6)	
**Disorders of amino acid metabolism**	12/19	34 years (16–39)	Urea cycle disorders and inherited hyperammonemias	14 (45)	12 (39)
			Organic acidurias	6 (17)	
			Disorders of branched-chain amino acid metabolism	3 (10)	
			Disorders of phenylalanine and tyrosine metabolism	3 (10)	
			Disorders of amino acid transport	2 (6)	
			Disorders of the metabolism of sulfur-containing amino acids and hydrogen sulfide	2 (6)	
			Disorders of glycine and serine metabolism	1 (3)	
**Disorders of carbohydrates**	17/9	34 years (16–64)	Disorders of glycogen metabolism	14 (54)	10 (38)
			Disorders of galactose and fructose metabolism	11 (42)	
			Not classified	1 (4)	

## Data Availability

Not applicable.
